# An Alarming Eastward Front of Cassava Mosaic Disease in Development in West Africa

**DOI:** 10.3390/v16111691

**Published:** 2024-10-29

**Authors:** Mariam Combala, Justin S. Pita, Michel Gbonamou, Alusaine Edward Samura, William J.-L. Amoakon, Bekanvié S. M. Kouakou, Olabode Onile-ere, Seydou Sawadogo, Guy R. Eboulem, Daniel H. Otron, John Steven S. Seka, Angela Eni, Cyrielle Ndougonna, Fidèle Tiendrébéogo

**Affiliations:** 1 UFR Biosciences, Université Félix Houphouët-Boigny, Abidjan 22 BP 582, Côte d’Ivoire; mariamcombala1988@gmail.com (M.C.); justin.pita@wave-center.org (J.S.P.); bekanviemarie@yahoo.fr (B.S.M.K.); danyotron452@gmail.com (D.H.O.); steveseka7@gmail.com (J.S.S.S.); 2 The Central and West African Epidemiology (WAVE) for Food Security Program, Pôle Scientifique et D’innovation, Université Félix Houphouët-Boigny, Bingerville 22 BP 582, Côte d’Ivoire; willamoakon@gmail.com (W.J.-L.A.); bodeoni@yahoo.com (O.O.-e.); seydousawadogo66@gmail.com (S.S.); guy.eboulem@wave-center.org (G.R.E.); angela.eni@wave-center.org (A.E.); cyrielle.ndougonna@wave-center.org (C.N.); 3 Institut de Recherche Agronomique de Guinée, Conakry BP 1523, Guinea; gbonamoum@gmail.com; 4 Department of Crop Protection, School of Agriculture and Food Sciences, Njala University, Njala Campus, Njala 1313, Sierra Leone; alusaine.samura@wave-center.org

**Keywords:** cassava, begomovirus, EACMV, EACMV-Ug, Guinea, Sierra Leone

## Abstract

Begomoviruses are a major threat to cassava production in Africa. Indeed, during the 1990s, the emergence of a recombinant begomovirus (East African cassava mosaic virus-Uganda, EACMV-Ug) resulted in crop devastation and severe famine in Uganda. In 2023, during a pre-survey of cassava farms at Forécariah, South-West Guinea, 22 samples showing peculiar cassava mosaic disease (CMD) symptoms were collected, and subsequent laboratory analysis confirmed the presence of EACMV-Ug in the samples. Deep analysis of DNA-A and DNA-B of the EACMV-Ug isolates from Guinea indicated that they are similar to those associated with the severe CMD epidemic in Uganda in the 1990s. Therefore, a country-wide survey was conducted throughout Guinea in April 2024 to evaluate the extent of the spread of EACMV-Ug in the country and to collect critical CMD epidemiological data. Findings showed a high whitefly population in Lower Guinea averaging 17 per plant; however, the data suggest a spread of EACMV-Ug via infected cuttings. High CMD incidence was found in Lower Guinea and Forest Guinea, whereas the highest CMD severity was observed in Forest Guinea (2.70 ± 0.06) and the lowest CMD severity was found in Middle Guinea (2.20 ± 0.05). Several cases of double and triple infections involving African cassava mosaic virus, East African cassava mosaic virus, East African cassava mosaic Cameroon virus, and EACMV-Ug were observed. EACMV-Ug was detected throughout Guinea, as well as from samples collected in 2022 in Kambia (Sierra Leone). The 63 accessions cultivated in Guinea that were assessed in this study were found susceptible to at least one of the viruses cited above. This study alerts us to an alarming situation in development in West Africa and provides scientific evidence to guide the rapid response needed to contain and stop the progression of EACMV-Ug in West Africa.

## 1. Introduction

Cassava (*Manihot esculenta* Crantz) is an important crop for more than 800 million people in Africa [1]. The starch-rich crop is used in many countries and plays an important role in food security. In Guinea, cassava is now the most consumed crop, ahead of rice. However, cassava cultivation faces several challenges that limit its production. These challenges include disruption from climate change, inappropriate farming practices, pests, fungal, bacterial, and viral diseases [2]. Of all these constraints, viral diseases are proving very difficult to control, unlike diseases that can be managed with fungicides, bactericides, or insecticides. 

In West Africa, cassava is affected by cassava mosaic disease (CMD), which can reduce cassava yield by up to 90% [1]. CMD-infected plants can easily be identified by the color and morphology of their leaves. Initially, infected leaves show a distinctive mosaic pattern with alternating yellow and green colors until symptoms become more severe and the leaves start to distort, ultimately leading to plant stunting. The disease is transmitted by an insect vector, the whitefly *Bemisia tabaci*; however, the use of cuttings originating from diseased plants is also an important means of dissemination of the disease [3,4]. CMD is caused by single-stranded DNA viruses belonging to the genus *Begomovirus* of the family *Geminiviridae*. Their genome consists of two pieces of DNA called DNA-A and DNA-B, where DNA-A contains the genes responsible for replication, virus transmission, and symptom expression, while DNA-B codes for the movement of the virus within the plant [5]. 

CMD is an endemic disease in Africa caused by nine begomovirus species “https://ictv.global/report/chapter/geminiviridae/geminiviridae/begomovirus (accessed on 2 September 2024)”. The two species that commonly infect cassava on the African continent are the *African cassava mosaic virus* (ACMV) and *East African cassava mosaic virus* (EACMV). They may be present in the plant alone or in a mixed infection. When both viruses are present in the same plant, they interact synergistically, leading to an increased severity of symptoms [6]. Mixed infections are a potential source of viral diversity and evolution due to frequent recombination resulting in more virulent strains than the original parents [6].

In the 1990s, the sudden emergence of a recombinant named East African cassava mosaic virus-Uganda (EACMV-Ug) caused a severe epidemic of CMD in Uganda, devastating cassava plantations and causing famine in the country [7]. EACMV-Ug is a recombinant between ACMV and EACMV, with part of the ACMV coat protein gene integrated into the EACMV genome [8]. The hard-to-control whitefly vectors and the exchange of cuttings from one region to another were key factors in the rapid spread of this virulent virus in East Africa. Since then, the virus has steadily spread from Uganda to other surrounding countries: Tanzania and Kenya [9], Democratic Republic of Congo (DRC) [10], Gabon [11], Equatorial Guinea [12], and Cameroon [13]. In West Africa, EACMV-Ug was first reported in Burkina Faso in 2009 by Tiendrébéogo et al. [14] but has not been found during recent surveys [4].

In Guinea, previous research in 2004 reported only ACMV in cassava samples tested [15]. Later on, cassava field surveys conducted in Guinea by Bah et al. [16] showed that ACMV and EACMCMV (East African cassava mosaic Cameroon virus) were infecting cassava in single or double infections. In 2023, peculiar CMD symptoms were reported in Forécariah in South-West Guinea, about 34 km from the Sierra Leone border. Molecular analysis confirmed that the plants exhibiting these symptoms were infected by EACMV-Ug, thus reporting for the first time the presence of this virus in Guinea [17].

Given the threat that this virus poses to cassava cultivation, this work was carried out to evaluate the extent of its spread in Guinea and Sierra Leone, given the existence of a busy highway from Forécariah to Sierra Leone, and to provide accurate epidemiological data to guide its containment and avoid the ignition of an eastward epidemic front of CMD from these countries to other West African countries.

## 2. Materials and Methods

### 2.1. Pre-Survey in Forécariah and Generation of Complete Genome Nucleotide Sequences of Cassava Mosaic Begomovirus (CMBs)

In 2023, a cassava field survey was conducted around Forécariah, Guinea, and 22 leaf samples presenting peculiar symptoms, as well as some symptomless leaves, were collected. The samples were tested using the primer pairs JSP001/JSP002, JSP001/JSP003 and VNF031F/VNF032R [17,18]. Based on PCR results, samples were selected for rolling circle amplification followed by Nanopore MinION sequencing as described by Ben Chehida et al. [19].

### 2.2. Country-Wide Epidemiological Survey

In April 2024, a cassava field survey was conducted in four regions of Guinea: Lower Guinea, Middle Guinea, Upper Guinea, and Forest Guinea. In 2022, a similar country-wide cassava field survey was also carried out in Sierra Leone. Fields were sampled approximately every 10 km, based on the harmonized protocol established by the Central and West African Virus Epidemiology Center [20]. The diagonal X method was used to visually assess the fields visited. A total of 15 plants were randomly selected along 2 diagonals (30 plants in total) and assessed. Geo-coordinates including longitude, latitude, and altitude were recorded for each survey site using a global positioning system (GPS). 

Epidemiological data collected using kobocollect included plant age, disease incidence in the field, severity of CMD symptoms, number of whiteflies per plant, type of infection (by whitefly or from infected cutting), and names of cassava varieties. In each field, up to five samples were selected based on symptom severity: very severe, severe, mild, symptomless, and others with particular symptoms. Each sample collected consists of lignified cassava cuttings and leaves taken from one plant. The cuttings were grown in a greenhouse for future experiments and the young leaves were placed in a labeled envelope for molecular testing. 

The mean incidence of CMD (Im) was calculated using the following formula:

Im = Number of diseased plants/Total number of plants assessed

**Figure 1:**
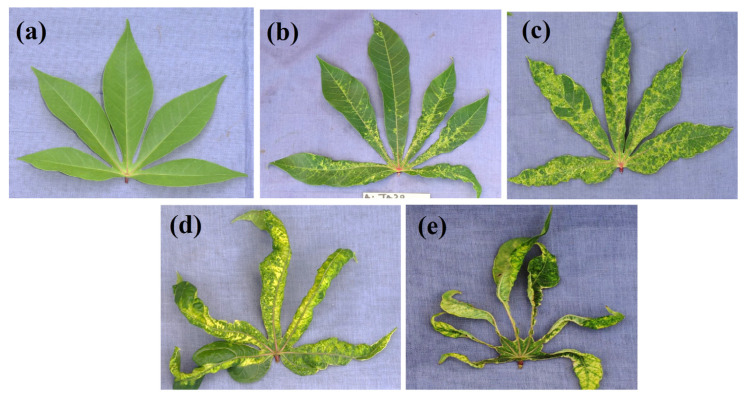
Symptoms of cassava mosaic disease observed on infected cassava leaves using a scale from 1 (no symptoms) to 5 (very severe symptoms): (**a** = score 1): leaf without mosaic symptoms; (**b** = score 2): leaf with chlorotic spots; (**c** = score 3): leaf with mosaic spots on the whole leaf and leaf twisting; (**d** = score 4): leaf with mosaic symptoms and leaf distortion on 2/3 of the leaf area; (**e** = score 5): leaf with mosaic symptoms and leaf distortion on 4/5 of the leaf area, and stunting of the whole plant [22,23].

The severity of CMD symptoms was scored by visual assessment using a scale of 1–5 [21], with 1 representing no symptoms and 5 representing very severe symptoms (Figure 1). The mean severity (Sm) was calculated using the following formula:

Sm = Σ scores of diseased plants/Total number of diseased plants

The infection mode was categorized as either whitefly-borne or cutting-borne. If only the apical leaves showed CMD symptoms with no symptoms observed on the lower leaves, the infection mode was considered to be whitefly-borne. If the lowest first leaves or all leaves of the plants showed CMD symptoms, the infection was considered to be cutting-borne [22]. The abundance of the whitefly population was determined by counting the number of adult whiteflies present on the five youngest apical leaves of the plants [21]. 

### 2.3. Molecular Characterization of CMBs

Total DNA was extracted from cassava leaf samples according to a modified version of the protocol described by Doyle and Doyle [24]. To perform the polymerase chain reaction (PCR), the DNA concentration of each sample was determined using a spectrophotometer (Eppendorf AG, Hamburg Germany) and adjusted to 150 ng/μL. Specific primer pairs were used to detect CMD viruses (Table 1). The PCR reaction mixture was prepared to a final volume of 25 μL as described by Amoakon et al. [23]. The reaction consisted of 1 × GoTaq reaction buffer (Promega, Madison, WI, USA), 0.625 U GoTaq polymerase (Promega), 0.4 μM of each primer, 0.2 mM dNTP (New England Biolabs), 1 mM MgCl_2_ (Promega). For all the primer pairs, amplification conditions consisted of an initial denaturation step at 94 °C for 4 min; 35 cycles of 94 °C for 1 min, 55 °C for 1 min, and 72 °C for 1 min; and a final extension at 72 °C for 10 min. A volume of 10 μL of amplified products was used for electrophoresis on a 1% agarose gel. The gel was then stained with ethidium bromide, visualized under UV light, and photographed using a gel imager (UV Transilluminator-26, VWR, Philadelphia, PA, USA).

### 2.4. Sequencing and Phylogenetic Analysis

PCR products were sequenced using the Sanger method (Genewiz, Germany). After cleaning the sequences using Geneious Prime 2024.0.7 software, the resulting contigs were subjected to BLASTn search in the NCBI database. Representative sequences of known cassava begomoviruses were downloaded from GenBank for phylogenetic analyses. Multiple sequence alignments of sequences obtained from this study, as well as representative cassava begomoviruses, were computed using the ClustalW algorithm with default settings. Relatedness between isolates was determined using the Maximum Likelihood Method with a general time reversible (GTR) model and 1000 bootstrap replicates. All analyses were conducted using MEGA 11.

### 2.5. Statistical Analysis

Epidemiological data were analyzed using R v.4.4.1 (R Core Team, 2024). Differences in CMD incidence, CMD mean severity, mode of infection, and whitefly abundance by sampling region were assessed using a generalized linear model (*glm*) with a likelihood-ratio test (chi-squared test or Fisher test in case of overdispersion). Post hoc analysis was computed using Tukey’s Pairwise test. Plots were generated with the ggplot2 package [30].

## 3. Results

### 3.1. Characterization of EACMV-Ug in Guinea 

Molecular analysis of the 22 samples collected in 2023, in Forécariah, revealed the presence of ACMV in single infection (14/22), EACMV-Ug in single infection (2/22), and the two viruses ACMV and EACMV-Ug in mixed infection (4/22). The remaining two samples were negative for all the primer pairs used. To confirm the presence of EACMV-Ug and obtain its complete genome nucleotide sequence, rolling circle amplification (RCA) was performed on selected samples followed by Nanopore MinION sequencing. A total of four full-length sequences were obtained. These included one DNA-A from EACMV-Ug (LC832863), one DNA-A from ACMV (LC832866), and two DNA-Bs from EACMV-Ug (LC832864; LC832865). Phylogenetic analysis of the sequences obtained confirmed that they are closely related to the sequences of the EACMV-Ug variant from Uganda. 

### 3.2. Analysis of EACMV-Ug Full-Length Genome Detected in Guinea

The DNA-A genome fragment of 2799 bp (LC832863) from Guinea shared 98.89% nucleotide identity with EACMV-Ug (HE814064) from Chad and 98.6% with EACMV-Ug (NC_004674) known as “Uganda2 Severe” strain from Uganda. The two DNA-B genome fragments of 2774 bp (LC832864) and 2777 bp (LC832865) from Guinea shared 99.9% of nucleotide identity amongst them and were both 97.73% identical to EACMV-Ug (KM885991) from Central African Republic. The two DNA-Bs shared 97.3% nucleotide identity with the DNA-B (NC_004676) known as the “Uganda3 Severe” strain.

Analysis of the sequences of the common region (CR) (186 bp) showed that our new DNA-A sequence (LC832863) and EACMV Uganda2 Severe (NC_004674) share 97.8% nucleotide identity. The same analysis between the CRs (186 bp) of EACMV-Ug DNA-A (LC832863) and EACMV-Ug DNA-Bs (LC832864 or LC832865) from Guinea showed that they share only 60.8% nucleotide identity. Similarly, using the same comparison method, we found 61.3% nucleotide identity between Uganda2 Severe DNA-A (NC_004674) and Uganda3 Severe DNA-B (NC_004676). 

### 3.3. CMD Incidence and Severity in Guinea 

**Figure 2:**
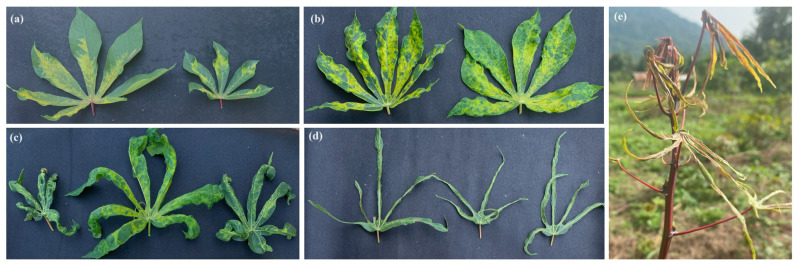
Symptoms of cassava mosaic disease observed in cassava fields in Guinea: (**a**) mild mosaic; (**b**) severe mosaic and slight deformation of leaves; (**c**) very severe mosaic, deformation and curling of leaves; (**d**) filiform leaves; (**e**) plant with filiform leaves.

**Figure 3:**
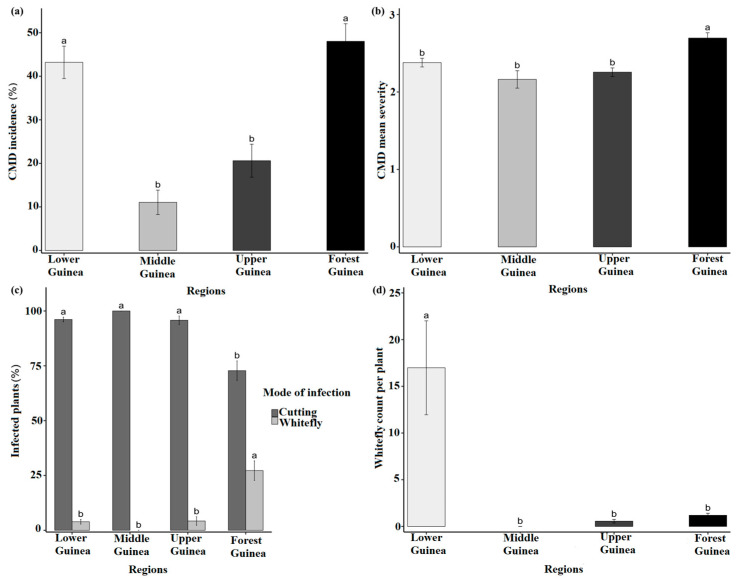
Epidemiological assessment of cassava mosaic disease (CMD) in Guinea: (**a**) mean incidence of CMD, (**b**) mean severity of CMD, (**c**) percentage of plants infected by cuttings or whiteflies; (**d**) number of whiteflies per plant. Error bars represent standard error (±SE); treatments with the same letter are not significantly different.

**Figure 4:**
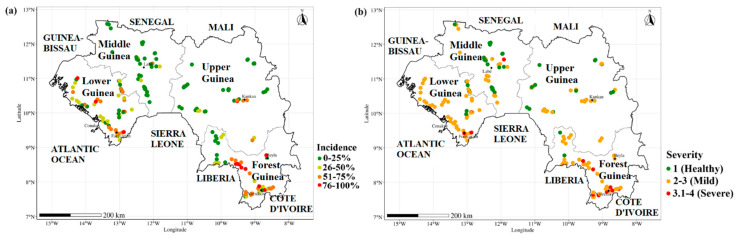
Variation in mean CMD incidence (**a**) and severity (**b**) in cassava fields in Guinea.

CMD symptoms including mild mosaic, severe mosaic, slight deformation of leaves, very severe mosaic and deformation, leaf curling, and filiform leaves were observed in cassava fields in Guinea (Figure 2). A total of 163 cassava fields were surveyed in four regions: Lower Guinea (48), Middle Guinea (35), Upper Guinea (38), and Forest Guinea (42). A comparison of the mean CMD incidence and symptom severity showed a highly significant difference between regions (*p* < 0.01). CMD incidence varied between regions with 48.02 ± 0.04% for Forest Guinea (the highest disease incidence) and 43.19 ± 0.04% in Lower Guinea, while Middle Guinea and Upper Guinea presented the lower CMD incidence rates at 11.05 ± 0.02% and 20.61 ± 0.04%, respectively (Figure 3a). The highest disease severity was recorded in Forest Guinea (2.70 ± 0.06). Regarding CMD severity, no significant difference was observed between Lower Guinea (2.40 ± 0.05), Middle Guinea (2.20 ± 0.05), and Upper Guinea (2.30 ± 0.05) (Figure 3b). Figure 4 shows the variation in mean CMD incidence and severity in the different regions of Guinea. CMD incidence and severity were higher in Forest Guinea and Lower Guinea.

### 3.4. Mode of Infection and Whitefly Abundance in Guinea

Statistical analysis showed a highly significant difference (*p* < 0.01) between regions in terms of infection mode and whitefly abundance. Overall, infection through cuttings was the most prevalent mode of propagation for the disease in all four regions. However, the proportion of cutting infection was 72.79 ± 0.04% in Forest Guinea, which is relatively low compared to the other regions where the proportions of cutting infection were 95.77 ± 0.02% (Upper Guinea), 96.12 ± 0.01% (Lower Guinea), and 100 ± 0.00% (Middle Guinea). The percentage of whitefly infection was relatively low in all the regions ranging from 0.00 ± 0.00% in Middle Guinea, 3.88 ± 0.01% in Lower Guinea, and 4.23 ± 0.02% in Upper Guinea with no significant difference between regions. In contrast, Forest Guinea recorded the highest value 27.21 ± 0.04% (Figure 3c). The number of whiteflies per plant ranged from 0 to 17. The highest value was observed in Lower Guinea (17 ± 5.02). Whiteflies were very rare in Middle Guinea, Upper Guinea had 0.56 ± 0.19, and 1.2 ± 0.21 was observed in Forest Guinea (Figure 3d). 

### 3.5. Detection of CMBs by PCR and Their Distribution Across Guinea

**Figure 5:**
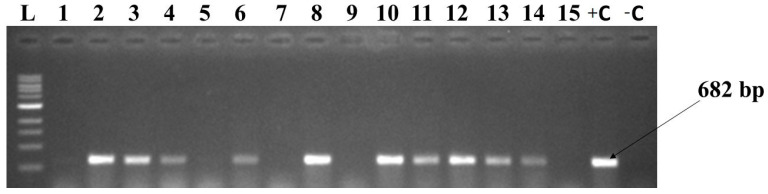
Gel electrophoresis (1% agarose) of the PCR products for detection of CMD. L = 1 kb DNA ladder, + C = positive control, −C = negative control.

African cassava mosaic virus (ACMV), East African cassava mosaic virus (EACMV), East African cassava mosaic Cameroon virus (EACMCMV), and East African cassava mosaic virus-Uganda (EACMV-Ug) were detected in cassava leaf samples collected in Guinea by PCR (Figure 5). A total of 393 samples were tested, including 120 from Lower Guinea, 62 from Middle Guinea, 90 from Upper Guinea, and 121 from Forest Guinea. The infection rate was 84.48% (332 infected/393 tested). In total, 61 samples were negative for the entire set of primers used for diagnostics out of the 393 samples tested (15.52%). The highest infection rate was observed in Forest Guinea (27.23%), followed by Lower Guinea (23.92%), Upper Guinea (20.36%), and Middle Guinea (12.98%) (Table 2). ACMV in single infection was predominant in all regions. EACMV in single infection and EACMV-Ug in single infection were only observed in Lower Guinea and in Forest Guinea. In contrast, EACMCMV in single infection was detected in all regions except in Lower Guinea. Interestingly, several instances of multiple infections were observed. These included ACMV+EACMV (4.07%), ACMV+EACMCMV (14.76%), ACMV+EACMV-Ug (10.947%), ACMV+EACMV+EACMV-Ug (0.51%), and ACMV+EACMCMV+EACMV-Ug (4.83%). The triple-infection ACMV+EACMV+EACMV-Ug was observed in Upper Guinea only (Table 2).

**Figure 6:**
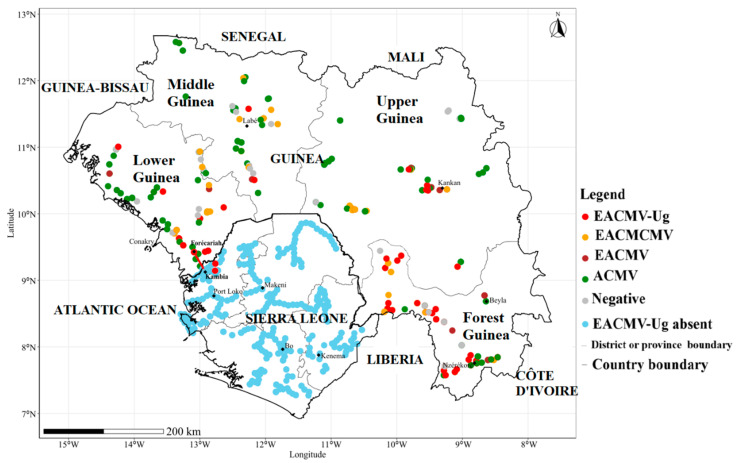
Distribution map of cassava mosaic begomoviruses (ACMV, EACMV, EACMCMV, and EACMV-Ug) in cassava fields in Guinea and distribution of EACMV-Ug in Sierra Leone.

The distribution map for the CMBs detected in this study (ACMV, EACMV, EACMCMV, and EACMV-Ug) is shown in Figure 6. The data confirm that EACMV-Ug, which was first detected in Forécariah in Lower Guinea, is present in the other three regions of Guinea.

Furthermore, since Forécariah is situated 34.8 km from Sierra Leone’s borders with Guinea and given the high traffic and trade existing between Forécariah and Sierra Leone, it was necessary to investigate a possible propagation of EACMV-Ug from Forécariah to Sierra Leone through exchanges of cassava planting materials. Fortunately, historical samples from the country-wide survey conducted in Sierra Leone in 2022 were well-preserved and were screened for the presence of EACMV-Ug.

Out of the 718 samples from Sierra Leone screened, only two were infected by EACMV-Ug. These EACMV-Ug-infected samples were from fields surveyed in the vicinity of Kambia, a town located about 11 km from the Guinean border (Figure 6).

### 3.6. Cassava Variety Range and Infecting Begomoviruses in Guinea

**Figure 7:**
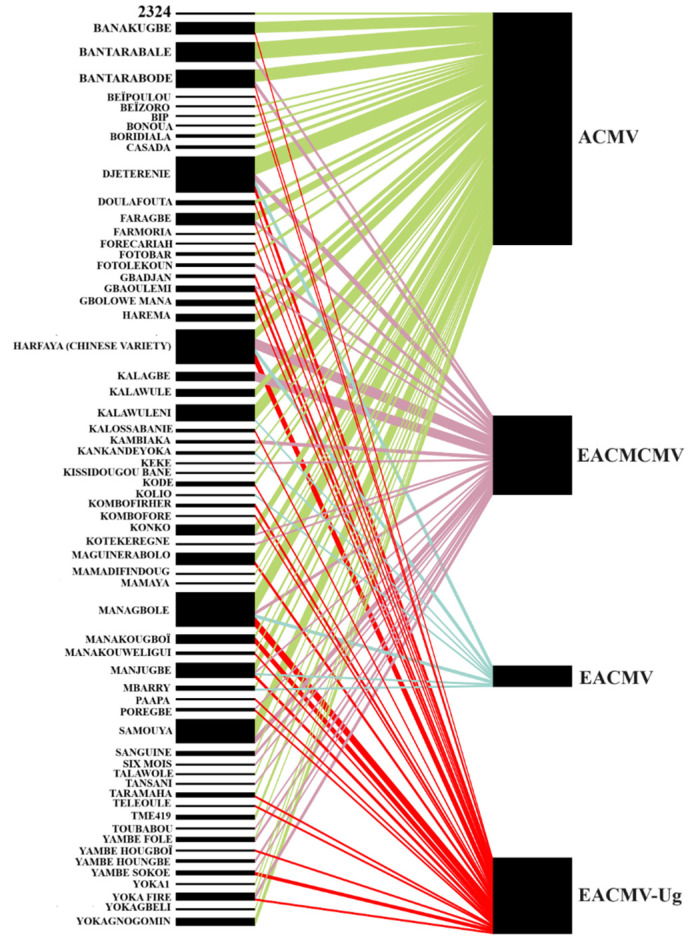
Sankey diagrams illustrating the affinities between cassava varieties and cassava mosaic begomoviruses identified in Guinea. The width of each node and the number of flow lines are proportional to the number of infected samples of the same cassava variety.

A total of 289 cassava varieties with known names were collected during the 2024 country-wide survey in Guinea. Begomoviruses were detected in the 63 varieties on the diagonal. Figure 7 shows the affinities between these cassava varieties and the CMBs detected in cassava fields in Guinea. All cassava varieties were infected by at least one begomovirus. In total, 68.25% (43/63) of the varieties were infected by ACMV and only 12.70% (8/63) with EACMV. EACMCMV was found in 38.10% of the varieties (24/63). Also, 42.85% (27/63) of the varieties were infected with the Ugandan variant EACMV-Ug. However, three cassava varieties, Managbole, Harfaya (Chinese variety), and Manakougboï appeared to be the most susceptible to EACMV-Ug infection (Figure 7). These varieties are grown in Lower Guinea and Forest Guinea, where EACMV-Ug incidence is the highest. 

### 3.7. Phylogenetic Relationships Between EACMV-Ug Coat Protein Genes

**Figure 8:**
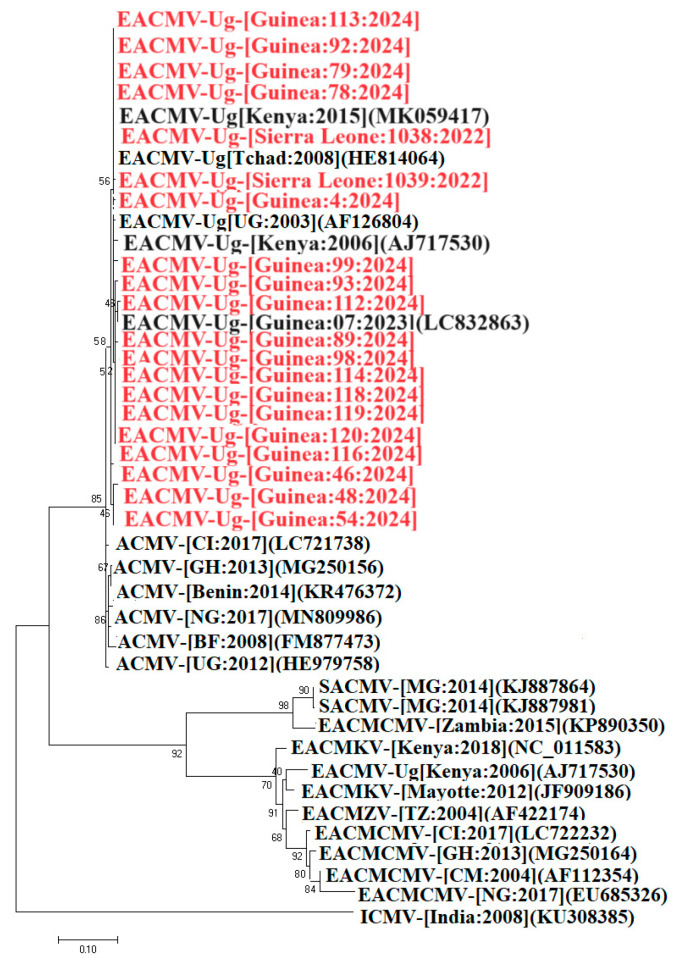
Maximum likelihood phylogenetic tree showing the relationships between Guinea isolates of East African cassava mosaic-Uganda virus (EACMV-Ug; 18 isolates), and diverse representative isolates of cassava mosaic begomoviruses. The tree is based on partial sequences for EACMCV-Ug (DNA-A coat protein) and rooted using Indian cassava mosaic virus (GenBank accession number, DNA-A: KU308385) as an outgroup. Sequences obtained in this study are in red while those in black were obtained from GenBank. Bootstrap analysis was performed with 1000 replicates.

Partial fragments of the coat protein (CP) genes of EACMV-Ug were amplified using primer pairs JSP001/JSP003 and WAVE177F/WAVE569R. A total of 21 CP sequences were used for the phylogenetic analysis. Nineteen of these sequences were from Guinea and the other two were from Sierra Leone (Kambia). Comparison of these sequences with those in the GenBank database showed that all the sequences obtained were closely related to the Ugandan EACMV-Ug variant. Indeed, the 19 sequences from Guinea shared 98.86% to 99.87% nucleotide identity with isolates from Kenya (MK059417; AJ717530), Uganda (AF126804), and Chad (HE814064). However, the two sequences from Sierra Leone shared 97.21% and 97.08% nucleotide identity with the isolate from Kenya (MK059417). The maximum likelihood model phylogenetic tree constructed using the alignment of EACMV-Ug CP sequences from Guinea and Sierra Leone shows that they are most closely related to isolates from Kenya, Uganda, and Chad (Figure 8). These results confirm that EACMV-Ug is circulating within Guinea and between Guinea and Sierra Leone.

## 4. Discussion

In this paper, we report two major findings: the detection of EACMV-Ug in Guinea and its spread beyond the country’s borders, highlighting the threat posed to neighboring countries. This virus was first reported in 2023 in Forécariah, a town located near the border with Sierra Leone [17]. It was found in single infection as well as in mixed infections with ACMV. Co-infection of both viruses could result in a synergistic interaction with severe impact on cassava fields, leading to the emergence of epidemics, as was the case in East Africa in the 1990s [12,14,26]. 

Phylogenetic analysis of the full-length nucleotide sequences of EACMV-Ug revealed a trans-replication between the DNA molecules circulating in Guinea and Sierra Leone, which are very similar to EACMV-Ug2 DNA-A and EACMV-Ug3 DNA-B described in Uganda [6]. According to these authors, the reassortant virus resulting from this association causes very severe CMD symptoms and was favored over the existing less severe EACMV-Ug1 strain. Moreover, the analysis of the common region of the EAMCV-Ug strain from Guinea and Sierra Leone indicated that it is closely related to the one associated with the Ugandan epidemic. This finding is worrisome for Guinea and its neighboring countries, given the way EACMV-Ug devasted cassava plantations in Uganda during the 1990s epidemic. To investigate these findings further, a country-wide cassava field survey was conducted in 2024 to evaluate the extent of the spread of EACMV-Ug across Guinea. This large-scale epidemiological survey showed a high incidence of CMD in Guinea. This incidence could largely be attributed to the continuous cultivation of susceptible cassava varieties and to the use of diseased planting materials [31]. Our results showed that CMD incidence varied greatly between regions. Forest Guinea and Lower Guinea recorded higher mean CMD incidence compared to Middle Guinea and Upper Guinea. Forest Guinea is located in the Eastern part of the country and borders western Côte d’Ivoire, which was found to be most impacted by CMD according to Kouakou et al. [20]. Considering the regular exchanges of planting materials between the two countries, there is a high risk of introducing EACMV-Ug into Côte d’Ivoire from Guinea. Therefore, via this communication, we are alerting the regional and national authorities to instigate a rapid response in Guinea and Sierra Leone, and the instauration of precautionary measures in Côte d’Ivoire. The high CMD incidence observed in Lower Guinea is indicative of the risks of CMD propagation to new areas. Such spread is largely the result of farmers exchanging planting materials without first considering their phytosanitary status [32]. The lowest CMD incidence found in Middle Guinea could be attributed to the high altitude characterizing this region. Indeed, according to Harimalala et al. [33] prevalence of CMD decreases with increasing altitude. In addition, the low incidence of CMD in this region could be a result of the common use of CMD-resistant cassava varieties compared to the other regions [4]. The significant prevalence of CMD observed in Lower Guinea and Forest Guinea can also be attributed to the fact that these two regions are characterized by intense agricultural activities, supported by frequent rainfall in these areas. In contrast, the regions of Middle Guinea and Upper Guinea, which are more conducive to livestock production, have a much lower incidence of CMD.

It is worth highlighting the positive correlation between the severe CMD symptoms and the high CMD incidence observed in Forest Guinea with the presence of EACMV-Ug. This was the hallmark of the severe Uganda CMD epidemic that led to the near-complete elimination of the most vulnerable cassava varieties [32,34]. Fortunately, a critical parameter of the devastating Uganda CMD epidemic is not at play here. The superabundant whitefly population, which was a key driver of the Ugandan epidemic, is not widespread in Guinea. The number of whiteflies was relatively low in Forest Guinea. However, our data indicate that the few whiteflies found in this region might be able to efficiently transmit CMD as the rate of whitefly infection was relatively high there compared to the other regions. On the other hand, the second high-risk region, Lower Guinea, where CMD incidence was also high, recorded some of the highest whitefly numbers reported in some countries in West Africa [35,36]. The abundance of whiteflies in Lower Guinea can be attributed to the proximity of cassava fields to vegetable crops. Due to the high rainfall climate in this region, farmers tend to prioritize food crops. These observations are consistent with those of Tiendrébéogo et al. [37], who demonstrated that vegetable crops serve as hosts for whiteflies.

The current situation is alarming because this is where we first discovered EACMV-Ug and it is now spread all over Guinea. Much attention must be given to controlling a whitefly population surge in this region to avoid any CMD outbreak in West Africa.

Molecular analysis of the samples collected during the 2024 survey in Guinea showed that the country is becoming a hot spot for cassava begomoviruses diversity in West Africa. Indeed, four begomoviruses, ACMV, EACMV, EACMCMV, and EACMV-Ug, were previously found in single, double, and triple infections in Madagascar [33]. To our knowledge, this is the first report of such multiple associations of cassava mosaic begomoviruses in cassava plants in West Africa. It reflects the importance of CMD pressure on cassava in Guinea and raises the question of the origin of such a wide diversity of CMBs in this country. ACMV was the most detected in all four regions of Guinea as observed in almost all sub-Saharan African countries where CMD occurs [4,35,38]. Although triple infections were found in all the regions in Guinea, Forest Guinea registered the highest number of plants infected by ACMV+EACMCMV+EACMV-Ug associated with very severe CMD. The synergistic action between the viruses involved in this triple infection probably contributed to the increased symptom severity as mentioned by Harimalala et al. [33]. Cases of triple ACMV+EACMV+EACMV-Ug infections were also reported in Burundi [39].

Since EACMV-Ug was reported in all four regions in Guinea, including at the border with Sierra Leone, historical samples from a survey conducted in 2022 in Sierra Leone were re-analyzed. As suspected, EACMV-Ug was found in Sierra Leone, precisely in Kambia, a location found 11 km from the border with Guinea. This result suggests a recent introduction from Guinea and an alarming eastward spread similar to the southward spread that occurred in Uganda during the CMD epidemic mentioned in several studies [40,41].

Another critical result to consider in this work is the detection of EACMV in Guinea for the first time. Previous molecular studies conducted in Guinea by Bah et al. [16] only detected ACMV and EACMCMV. The fact that EACMV and EACMV-Ug in single infections were only observed in Lower Guinea and Forest Guinea suggests that the two viruses might have been introduced into the country in a similar way and probably at the same time.

Phylogenetic analysis of EACMV-Ug DNA-A sequences obtained in this study showed that they were closely related to those from East and Central Africa. In addition, the Guinean and Sierra Leonian EACMV-Ug sequences contain the same ACMV recombinant fragment in their CP. These findings along with the fact that it is the same reassortant virus EACMV-Ug2 DNA-A + EACMV-Ug3 DNA-B that is circulating in Guinea and Sierra Leone, suggest that this virus was probably introduced into Guinea from East or Central African countries via infected cassava planting materials.

During the 2024 field survey, the names of the cassava varieties cultivated in Guinea were recorded. More than 60 varieties are currently grown throughout the country. These are generally local varieties as mentioned by Okao-Ohuia et al. [15]. These cassava varieties were all found to be susceptible to CMD with ACMV detected in all varieties, although some were more susceptible than others. Only eight varieties were infected by EACMV while EACMCMV and EACMV-Ug infected around 40% of cassava varieties found in the fields visited. Taken together, these results advocate for the urgent deployment of CMD management strategies in the region. 

## 5. Conclusions

This study confirms that CMD is an important constraint to cassava production in Guinea. The eastern and coastal regions were most affected by the disease. The spread of CMD throughout the country was linked to the use of infected planting material. Our findings described for the first time an unprecedented diversity of four CMBs, ACMV, EACMV, EACMCMV, and EACMV-Ug, infecting diverse local cassava varieties in Guinea. The identification of EACMV-Ug throughout the four Guinean regions and at the border with Sierra Leone presents a serious threat to cassava production in West Africa. This discovery suggests the need for an urgent rapid intervention through the implementation of control strategies by national and regional cassava stakeholders in West Africa. We recommend that cassava field surveys be conducted urgently to re-assess the spread of EACMV-Ug not only in Guinea but also in neighboring countries. Additionally, efforts must be made to raise awareness of the impact of CMD among farmers, extension officers, and decision makers, and to train stakeholders on CMD symptoms recognition and best practices. Finally, the strict implementation of phytosanitary procedures including seed certification, and the deployment of resistant varieties must be considered to halt the spread of EACMV-Ug in the West Africa region.

## Figures and Tables

**Table 1:** List of primers used for PCR detection of cassava begomoviruses from cassava leaf samples.

Primer Name	Primer Sequence	Size (bp)	Virus Detected (Target Region)	References
WAVE-177F	TCGAAGCCCAGATGTCCCTA	393	ACMV/EACMCMV/EACMV/ EACMV-Ug/AV1 (CP)	Combala et al. [25]
WAVE-569F	CCACCAACAACAGTGGCATG			
JSP001F	ATGTCGAAGCGACCAGGAGAT	783	ACMV/AV1 (CP)	Pita et al. [26]
JSP002R	TGTTTATTAATTGCCAATACT			
WAVE-AA508F	AAGGCCCATGTAAGGTCCAG	800	ACMV/AV1/AC3	Combala et al. [25]
WAVE-AA1307R	GAAGGAGCTGGGGATTCACA			
WAVE-AA370F	ACAGCCCATACAGGAACCGT	1000	ACMV/AV1/AC3	Combala et al. [25]
WAVE-AA1369R	CGACCATTCCTGCTGAACCA			
ACMVBF	TCGGGAGTGATACATGCGAAGGC	628	ACMV/BV1/BC1	Matic et al. [27]
ACMVBR	GGCTACACCAGCTACCTGAAGCT			
WAVE-AB177F	GATCTGCGGGCCTATCGAAT	800	ACMV/BV1	Combala et al. [25]
WAVE-AB977R	TTCACGCTGTGCAATACCCT			
WAVE-AB982F	TTCGTGTCATCTGCAGGAGA	800	ACMV/BV1/BC1	Combala et al. [25]
WAVE-AB1781R	GTACCATGGCAGCTGCTGTA			
JSP001F	ATGTCGAAGCGACCAGGAGAT	780	EACMV/AV1 (CP)	Pita et al. [26]
JSP003R	CCTTTATTAATTTGTCACTGC			
CMBRepF	CRTCAATGACGTTGTACCA	650	EACMV/AC1	Alabi et al. [28]
EACMVRepR	GGTTTGCAGAGAACTACATC			
VNF031F	GGATACAGATAGGGTTCCCAC	~560	EACMCMV/AC2/AC3	Fondong et al. [18]
VNF032R	GACGAGGACAAGAATTCCAAT			
WAVE-EA1875F	TGTACCAGGCGTCGTTTGAA	800	EACMV/AC1	Combala et al. [25]
WAVE-EA2674R	TGTCCCCCGATCCAAAACG			
EAB555F	TACATCGGCCTTTGAGTCGCATGG	544–560	EACMV/BC1/CR	Ndunguru et al. [29]
EAB555R	CTTATTAACGCCTATATAAACACC			
WAVE-EB1869F	TTCCAAGGGGAGGGTTCTGA	800	EACMV/BC1	Combala et al. [25]
WAVE-EB2694R	TGCTCTCGCCTCTCTCTTCT			
WAVE-EB845F	CGTGTATGGCATGCCTAGGT	1000	EACMV/BV1/BC1	Combala et al. [25]
WAVE-EB1847R	CTCCAACGTCATAGAAGGCGT			

**Table 2:** Cassava begomovirus combinations detected in Guinea by region.

Regions					
Viruses Detected	Lower Guinea (%)	Middle Guinea (%)	Upper Guinea (%)	Forest Guinea (%)	Total (%)
ACMV	54	34	52	42	182
	(13.74)	(8.65)	(13.23)	(10.69)	(46.31)
EACMV	2	0	0	1	3
	(0.51)	(0.00)	(0.00)	(0.25)	(0.76)
EACMCMV	0	1	3	1	5
	(0.00)	(0.25)	(0.76)	(0.25)	(1.27)
EACMV-Ug	3	0	0	1	4
	(0.76)	(0.00)	(0.00)	(0.25)	(1.02)
ACMV+EACMV	4	1	6	5	16
	(1.02)	(0.25)	(1.53)	(1.27)	(4.07)
ACMV+EACMCMV	15	13	12	18	58
	(3.82)	(3.31)	(3.05)	(4.58)	(14.76)
ACMV+EACMV-Ug	15	1	3	24	43
	(3.82)	(0.25)	0.76	6.11	10.94
ACMV+EACMV+EACMV-Ug	0	0	2	0	2
	(0.00)	0.00	(0.51)	(0.00)	(0.51)
ACMV+EACMCMV+EACMV-Ug	1	1	2	15	19
	(0.25)	(0.25)	(0.51)	(3.82)	(4.83)
Total infected samples	94	51	80	107	332
	(23.92)	(12.98)	(20.36)	(27.23)	(84.48)
Negative samples	26	11	10	14	61
	(6.62)	(2.80)	(2.54)	(3.56)	(15.52)
Total	120	62	90	121	393
	(30.53)	(15.78)	(22.90)	(30.79)	(100.00)

ACMV: African cassava mosaic virus; EACMV: East African cassava mosaic virus; EACMCMV: East African cassava mosaic Cameroon virus; EACMV-Ug: East African cassava mosaic virus-Uganda.

## Data Availability

Genomic sequences of begomoviruses ACMV DNA-A, EACMV-Ug DNA-A, EACMV-Ug DNA-B, and EACMV-Ug DNA-B are deposited in the NCBI GenBank under the accession numbers LC832866, LC832863, LC832863, LC832865. The dataset supporting the findings, and the results of this study are provided in the manuscript.

## References

[1] Vernier P., Boni N’Zué N.Z.-R. (2018). Le Manioc, Entre Culture Alimentaire et Filière Agro-Industrielle Quae CTA Presses Agronomiques de Gembloux. Editions Qua, CTA.

[2] He D.C., Zhan J.S., Xie L.H. (2016). Problems, challenges and future of plant disease management: From an ecological point of view. J. Integr. Agric..

[3] Njoroge M.K., Mutisya D.L., Miano D.W., Kilalo D.C. (2017). Whitefly species efficiency in transmitting cassava mosaic and brown streak virus diseases. Cogent Biol..

[4] Soro M., Tiendrébéogo F., Pita J.S., Traoré E.T., Somé K., Tibiri E.B., Néya J.B., Mutuku J.M., Simporé J., Koné D. (2021). Epidemiological assessment of cassava mosaic disease in Burkina Faso. Plant Pathol..

[5] Bull S.E., Briddon R.W., Sserubombwe W.S., Ngugi K., Markham P.G., Stanley J. (2006). Genetic diversity and phylogeography of cassava mosaic viruses in Kenya. J. Gen. Virol..

[6] Pita J.S., Fondong V.N., Sangare A., Otim-Nape G.W., Ogwal S., Fauquet C.M. (2001). Recombination, pseudorecombination and synergism of geminiviruses are determinant keys to the epidemic of severe cassava mosaic disease in Uganda. J. Gen. Virol..

[7] Legg J.P., Owor B., Sseruwagi P., Ndunguru J. (2006). Cassava Mosaic Virus Disease in East and Central Africa: Epidemiology and Management of A Regional Pandemic. Adv. Virus Res..

[8] Zhou X., Liu Y., Calvert L., Munoz C., Otim-nape G.W., Robinson D.J., Harrison B.D. (2017). Evidence that DNA-A of a geminivirus associated with severe cassava mosaic disease in Uganda has arisen by interspecific recombination. J. Gen. Virol..

[9] Were H.K., Winter S., Maiss E. (2004). Variations and taxonomic status of begomoviruses causing severe epidemics of cassava mosaic disease in Kenya, Uganda, and Democratic Republic of the Congo. Dis. Viroid.

[10] Neuenschwander P., Hughes J.A., Ogbe F., Ngatse J.M., De J.P.L. (2002). Occurrence of the Uganda variant of East African cassava mosaic virus (EACMV-Ug) in western Democratic Republic of Congo and the Congo Republic defines the westernmost extent of the CMD pandemic in East/Central Africa. Plant Pathol..

[11] Legg J.P., Ndjelassili F., Okao-okuja G. (2004). First report of cassava mosaic disease and cassava mosaic geminiviruses in Gabon. Plant Pathol..

[12] Valam-zango A., Zinga I., Hoareau M., Mvila A.C., Semballa S., Lett J.M. (2015). First report of cassava mosaic geminiviruses and the Uganda strain of East African cassava mosaic virus (EACMV-UG) associated with cassava mosaic disease in Equatorial Guinea. New Dis. Rep..

[13] Akinbade S.A., Hanna R., Njukwe E., Kuate A.F. (2010). First report of the East African cassava mosaic virus Uganda (EACMV-UG) infecting cassava (*Manihot esculenta*) in Cameroon. New Dis. Rep..

[14] Tiendrébéogo F., Lefeuvre P., Hoareau M., Traoré V.S.E., Barro N., Reynaud B., Traoré A.S. (2009). Occurrence of East African cassava mosaic virus -Uganda (EACMV-UG) in Burkina Faso. Plant Pathol..

[15] Okao-Okuja G., Legg J.P., Traore L., Jorge M.A. (2004). Viruses Associated with Cassava Mosaic Disease in Senegal and Guinea Conakry. J. Phytopathol..

[16] Bah E.S., Bamkefa B.A., Winter S., Dixon A.G.O. (2011). Distribution and current status of cassava mosaic disease and begomoviruses in Guinea. Afr. J. Root Tuber Crops.

[17] Combala M., Pita J.S., Tiendrebeogo F., Gbonamou M., Eni A.O. (2024). First Report of East African cassava mosaic virus-Uganda (EACMV-Ug) infecting cassava in Guinea, West Africa. New Dis. Rep..

[18] Fondong V.N., Pita J.S., Rey M.E.C., Kochko A., Beachy R.N., Fauquet C.M. (2000). Evidence of synergism between African cassava mosaic virus and new double-recombination geminivirus infecting cassava in Cameroon. J. Gen. Virol..

[19] Chehida S.B., Filloux D., Fernandez E., Moubset O., Hoareau M., Julian C., Blondin L., Lett J., Roumagnac P., Lefeuvre P. (2021). Nanopore Sequencing Is a Credible Alternative to Recover Complete Genomes of Geminiviruses. Microorganisms.

[20] Kouakou B.S.M., Yoboué A.A.N., Pita J.S., Mutuku J.M., Otron D.H., Kouassi N.K., Kouassi K.M., Vanié-Léabo L.P.L., Ndougonna C., Zouzou M. (2024). Gradual Emergence of East African cassava mosaic Cameroon virus in Cassava Farms in Côte d’Ivoire. Agronomy.

[21] Hahn S.K., Terry E.R., Leuschner K. (1980). Cassava for cassava resistance disease. Euphytica.

[22] Sseruwagi P., Sserubombwe W.S., Legg J.P., Ndunguru J., Thresh J.M. (2004). Methods of surveying the incidence and severity of cassava mosaic disease and whitefly vector populations on cassava in Africa: A review. Virus Res..

[23] Amoakon W.J.L., Yoboué A.A.N., Pita J.S., Mutuku J.M., N’Zué B., Combala M., Otron D.H., Koné M., Kouassi N.K., Sié R. (2023). Occurrence of cassava mosaic begomoviruses in national cassava germplasm preserved in two agro-ecological zones of Ivory Coast. Plant Pathol..

[24] Doyle J.J., Doyle J.L. (1987). A rapid DNA isolation procedure for small quantities of fresh leaf tissue. Phytochem. Bull..

[25] Combala M., Tibiri B.E., Pita J.S., Tiendrébéogo F., Eni A. (2024). Improving the cassava mosaic Begomoviruses diagnostic in Central and West Africa using Oxford nanopore technology. Sci. Rep..

[26] Pita J.S., Fondong V.N., Sangaré A., Kokora R.N.N., Fauquet C.M. (2001). Genomic and biological diversity of the African cassava geminiviruses. Euphytica.

[27] Matic S., Pais Da Cunha A.T., Thompson J.R., Tepfer M. (2012). An analysis of viruses associated with cassava mosaic disease in three Angolan provinces. J. Plant Pathol..

[28] Alabi O.J., Kumar P.L., Naidu R.A. (2008). Multiplex PCR for the detection of African cassava mosaic virus and East African cassava mosaic Cameroon virus in cassava. J. Virol. Methods.

[29] Ndunguru J., Legg J.P., Aveling T.A.S., Thompson G., Fauquet C.M. (2005). Molecular biodiversity of cassava begomoviruses in Tanzania: Evolution of cassava geminiviruses in Africa and evidence for East Africa being a center of diversity of cassava geminiviruses. Virol. J..

[30] Wickham H. (2016). Getting Started with ggplot2. ggplot2: Elegant Graphics for Data Analysis.

[31] Chikoti P.C., Peter S., Mulenga R.M., Tembo M. (2019). Cassava mosaic disease: A review of a threat to cassava production in Zambia. J. Plant Pathol..

[32] Anani J., Serge S., Pita J.S., Hound S.E. (2022). Cassava mosaic disease (CMD) in Benin: Incidence, severity and its whitefly abundance from field surveys in 2020. Crop Prot..

[33] Harimalala M., Chiroleu F., Giraud-Carrier C., Hoareau M., Zinga I., Randriamampianina J.A., Velombola S., Ranomenjanahary S., Andrianjaka A., Reynaud B. (2015). Molecular epidemiology of cassava mosaic disease in Madagascar. Plant Pathol..

[34] Thresh J.M., Otirn-nape D.F.G.W. (1994). Effects of African cassava mosaic geminivirus on the yield of cassava. Trop. Sci..

[35] Eni A.O., Efekemo O.P., Onile-ere O.A., Pita J.S. (2021). South West and North Central Nigeria: Assessment of cassava mosaic disease and field status of African cassava mosaic virus and East African cassava mosaic virus. Ann. Appl. Biol..

[36] Houngue J.A., Pita J.S., Habib G., Cacaï T., Zandjanakou-tachin M., Abidjo E.A.E., Ahanhanzo C. (2018). Survey of farmers’ knowledge of cassava mosaic disease and their preferences for cassava cultivars in three agro-ecological zones in Benin. J. Ethnobiol. Ethnomed..

[37] Tiendrébéogo F. (2010). Caractérisation et Aspects Epidémiologiques des Begomovirus Infectant les Plantes Maraîchères et le Manioc au Burkina Faso.

[38] Abubakar M., Singh D., Keta J.N. (2019). Cassava Mosaic Disease and Associated Gemini Viruses in Bauchi State, Nigeria: Occurrence and Distribution. Am. J. Plant Biol..

[39] Busogoro J.P., Masquellier L., Kummert J., Dutrecq O., Lepoivre P., Jijakli M.H. (2008). Application of a Simplified Molecular Protocol to Reveal Mixed Infections with Begomoviruses in Cassava. J. Phytopathol..

[40] Were H.K., Winter S., Maiss E. (2004). Occurrence and distribution of cassava begomoviruses in Kenya. Ann. Appl. Biol..

[41] Zhou X., Robinson D.J., Harrison B.D. (1998). Types of variation in DNA-A among isolates of East African cassava mosaic virus from Kenya, Malawi and Tanzania. J. Gen. Virol..

